# Bridging assessment, biomarkers, and interventions: progress in muscle health research

**DOI:** 10.3389/fresc.2026.1874273

**Published:** 2026-06-25

**Authors:** Dustin J. Oranchuk, Jason M. DeFreitas, André R. Nelson, Michael O. Harris-Love

**Affiliations:** 1 Muscle Morphology, Mechanics, and Performance Laboratory, School of Medicine, University of Colorado Anschutz Medical Campus, Aurora, CO, United States; 2 Department of Physical Medicine and Rehabilitation, University of Colorado Anschutz Medical Campus, Aurora, CO, United States; 3 Aging, Muscle, and Performance Laboratory, Department of Human Physiology and Nutrition, University of Colorado Colorado Springs, Colorado Springs, CO, United States; 4 Neural Health Research Laboratory, Falk College, Syracuse University, Syracuse, NY, United States; 5 Institute for Health and Sport, Victoria University, Melbourne, VIC, Australia; 6 Eastern Colorado VA Geriatric Research, Education, and Clinical Center, Aurora, CO, United States

**Keywords:** clinical populations, exercise, functional outcomes, muscle quality, muscle strength, precision medicine, sarcopenia, ultrasound imaging

## Abstract

The concept of ‘muscle health’ is increasingly recognized as a central determinant of physical function, metabolic regulation, and disease resilience, yet its clinical integration remains fragmented by inconsistent definitions and measurement approaches. This Perspective synthesizes insights from the Research Topic ‘*Advancing Muscle Health: From Technical and Clinical Research to Practice’,* which brings together nine contributions spanning assessment technologies, biomarkers, clinical populations, and interventions. Collectively, these works illustrate a field transitioning from isolated advances toward more integrated, clinically meaningful frameworks. Emerging ultrasound-based methods demonstrate how improved reliability and automation may enable scalable muscle assessment, while biomarker studies highlight both the promise and limitations of metabolomic, functional, and surrogate metrics in capturing the systemic nature of muscle health. Evidence from neurological, vascular, and oncological contexts reinforces that muscle is not only an outcome of disease, but a key modifier of disease progression and risk. Across these domains, exercise, particularly resistance-based and multimodal approaches, continues to emerge as a key, yet under-implemented, strategy. Despite this progress, critical gaps remain. The field lacks longitudinal, diverse-cohort data, standardized measurement frameworks, and robust integration of emerging technologies such as multi-omics and artificial intelligence. Moving forward, advancing muscle health will require interdisciplinary, translational approaches that align mechanistic insight with clinical application, enabling precise phenotyping and scalable interventions. Bridging these gaps is essential to move muscle health from a research construct to a core component of routine clinical care and public health strategy.

## 1 Introduction

**Figure 1:**
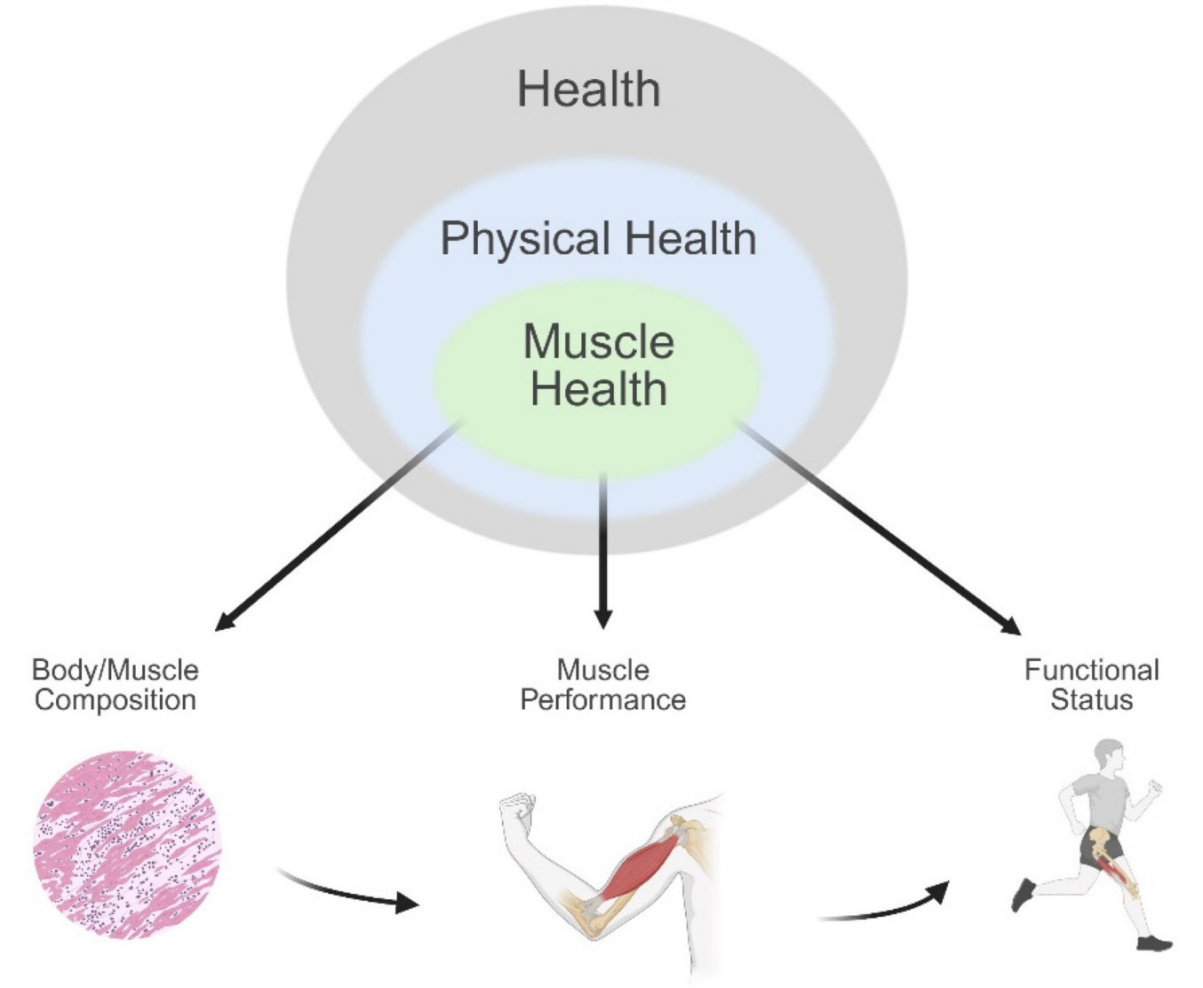
Proposed conceptual model of ‘muscle health’, positioned as an aspect of physical health and informed by the international classification of functioning, disability and health (ICF) framework. Adapted from Boncella et al., 2025 (1).

Muscle health is increasingly recognized as central to physical capability (e.g., mobility status), quality of life, overall well-being, and healthcare costs (1, 2). The widespread use of muscle health as a clinical research outcome is hindered by the lack of a formal definition and by its multidimensional nature. Recent work (1) has proposed a framework for muscle health that includes body/muscle tissue composition, muscle performance, and functional status (Figure 1**)**.

However, even within this framework, muscle dysfunction may stem from intrinsic alterations within the muscle itself (e.g., changes in muscle morphology, architecture, contractile properties, or metabolic function), from neurological dysfunction affecting muscle activation, or from a combination of both. The Research Topic, “Advancing Muscle Health: From Technical and Clinical Research to Practice,” was conceived to link foundational technical innovations, clinical investigations, and real-world applications. The Research Topic Editors sought contributions ranging from novel assessment frameworks and viable biomarkers of age- and disease-related muscle changes to the reliability of new evaluation methods, and interventions to enhance muscle function. The topic encouraged a diverse range of submissions, including original research, reviews, and technical papers, to foster translation. The nine accepted papers (3–11), summarized in Table 1, collectively provide multifaceted insights into these areas, demonstrating progress in diagnostic tools, predictive models, disease-specific impacts, and therapeutic strategies. This ‘Perspective’ synthesizes the themes of this Research Topic collection, highlighting how these works advance muscle health in the context of relevant research, and identifying challenges that should be addressed in the future.

**Table 1:** Overview of the ‘advancing muscle health: From technical and clinical research to practice’ special topic.

Authors, Journal & Section (by publication date)	Article Title	Article Type & Subtype	Bottom line
Morretti et al., 2025 (9) Frontiers in Rehabilitation Science Rehabilitation for Musculoskeletal Conditions	Physical exercise for primary sarcopenia: an expert opinion	Review: Opinion	Exercise is the cornerstone therapy for sarcopenia, with multimodal programs combining resistance, aerobic, and balance training providing the greatest overall benefits for muscle and functional outcomes.
Luera et al., 2025 (8) Frontiers in Rehabilitation Science Rehabilitation for Musculoskeletal Conditions	Comparison of manual and semi-automated algorithm for measuring architectural features during different isometric knee extension intensities: a reliability and comparative study in novice raters	Original Research: Technical	Semi-automated algorithms provide superior intra- and inter-rater reliability compared to manual ultrasound analysis, reducing user-dependent variability in muscle architecture assessment.
Hu et al., 2025 (3) Frontiers in Endocrinology Systems Endocrinology	Relationship between muscle quality index and urinary incontinence among U.S. population: evidence from NHANES 2011 to 2014	Original Research: Secondary analysis	Lower muscle quality is associated with a higher risk of urinary incontinence, highlighting muscle function as a modifiable target for prevention and management.
Edwards, Lulik-Kuryllo & Batra 2025 (7) Frontiers in Rehabilitation Science Rehabilitation for Musculoskeletal Conditions	The impact of second-generation androgen receptor pathway inhibitors on skeletal muscle morphology and strategies to mitigate their effects in prostate cancer patients	Review: Narrative	Androgen deprivation combined with ARPIs accelerates muscle deterioration in prostate cancer patients, highlighting the need to integrate exercise as part of standard supportive care.
Li et al., 2025 (4) Frontiers in Medicine Geriatric Medicine	The synergy between life's essential 8 and muscle strength on cardiovascular disease risk	Original Research: Biobank Analysis	Low muscle strength substantially increases cardiovascular disease risk primarily in the presence of poor overall cardiovascular health, indicating that muscle strength acts as a critical modifier that amplifies or mitigates lifestyle-related risk.
Rousseau et al., 2025 (5) Frontiers in Medicine Intensive Care Medicine and Anesthesiology	Serum acylcarnitines profile at ICU discharge to predict mid-term muscle outcomes: an exploratory study	Original Research: Brief Report	Acylcarnitine profiles alone fail to predict post-ICU muscle recovery, highlighting the need for multimodal approaches combining metabolic, functional, and clinical indicators.
Yan et al., 2025 (6) Frontiers in Psychiatry Schizophrenia	Ultrasonographic assessment of thenar muscles for diagnosing sarcopenic obesity in patients with schizophrenia	Original Research: Cross-sectional	Sarcopenic obesity in schizophrenia is characterized by reduced muscle thickness and increased echo intensity, and can be efficiently detected using thenar ultrasound with clinically relevant associations to adverse outcomes.
Shin & Lee 2026 (10) Frontiers in Medicine Geriatric Medicine	Cross-sectional comparison of lower-limb muscle strength and contractile properties according to Parkinson's disease and sarcopenia status	Original Research: Cross-sectional	Individuals with both Parkinson's disease and sarcopenia experience greater impairments in strength and muscle function, underscoring the need for integrated assessment and targeted rehabilitation strategies.
Pan et al., 2026 (11) Frontiers in Endocrinology Clinical Diabetes	Association between creatinine-to-body weight ratio and incident prediabetes in Chinese adults: a large-scale retrospective cohort study	Original research: Retrospective cohort	Low creatinine-to-body weight ratio identifies individuals at increased risk of prediabetes, highlighting muscle mass as a key and modifiable determinant of metabolic health.

## 2 Technological advancements in muscle assessment

A cornerstone of improving muscle health is refining non-invasive, reliable tools to evaluate muscle structure, composition, and function. Thus, ultrasound-based approaches are attractive because they are non-invasive, portable, relatively inexpensive, radiation-free, and capable of providing rapid assessments of both muscle quantity and muscle quality, making them particularly well suited for clinical applications. Recent work in this special issue highlights two complementary advances: improving the reproducibility of ultrasound-derived measurements and extending their utility to populations in whom conventional assessments may be impractical (6, 8).

Luera et al. (8) examined whether manual vs. semi-automated analysis affected the reliability of vastus lateralis architectural measurements during isometric knee extension in healthy, resistance-trained adults across different contraction intensities. Their findings showed that semi-automated analysis produced consistently stronger intra- and inter-rater reliability for fascicle length, pennation angle, and muscle thickness than manual measurement, particularly among novice raters. Importantly, a substantial body of work has already demonstrated the acceptable reliability of B-mode musculoskeletal ultrasound (12–16) across healthy, physically active, resistance-trained, older adult, and clinical populations, including studies evaluating automated and semi-automated image analysis approaches (17, 18). However, the authors observed that the reliability of the semi-automated algorithm was greater during active muscle contraction. This condition introduces additional image complexity and has been unexplored in prior investigations. Additionally, Luera et al. (8) extend this literature by incorporating novice raters, thereby addressing a critical and often underexplored translational barrier. This suggests that automation may help reduce one of the major barriers to broader adoption of ultrasound-based muscle assessment: dependence on analyst expertise. At the same time, the observed bias between manual and semi-automated outputs indicates that improved reproducibility should not be conflated with validity. Further work is still needed to determine how well these methods generalize across muscles, raters, and clinical populations.

Yan et al. (6) approached the problem from a different angle by testing whether ultrasonographic assessment of the thenar muscles could help identify sarcopenic obesity in patients with schizophrenia, a population characterized by high metabolic risk, reduced physical activity, and increased vulnerability to poor muscle health. Schizophrenia is associated with elevated burdens of sarcopenia, while the broader muscle ultrasound literature suggests that combining indices of muscle quantity and quality improves screening utility for sarcopenia-related phenotypes (19, 20). Consistent with this, Yan et al. (6) found that patients with sarcopenic obesity exhibited lower thenar muscle thickness and higher echogenicity (a proxy for intramuscular adipose and/or fibrotic tissue), and that a model combining right thenar thickness, echogenicity, and sex achieved good discriminatory performance (AUC=0.81). These findings are notable not only because they support ultrasound as a practical bedside screening modality, but also because they extend the field beyond traditional lower-limb muscle sites and into a high-risk psychiatric population (21, 22).

Taken together, these studies underscore that the future of muscle ultrasound likely depends on combining technical standardization with clinically meaningful composite metrics. This interpretation is consistent with literature suggesting that ultrasound has promise for sarcopenia and muscle health assessment, but that its diagnostic performance is strengthened when measures of muscle quantity and quality are integrated. Accordingly, the next step for the field is not simply the wider adoption of ultrasound, but the development of standardized acquisition protocols, validated analytic approaches, and population-specific thresholds that enable translation into clinical practice.

## 3 Biomarkers and predictors of muscle outcomes

Muscle health is not only a structural or functional construct, but also a systemic and metabolically integrated phenotype, reflected in circulating metabolites, composite indices, and surrogate markers of muscle mass and quality (1). The identification of clinically meaningful biomarkers of muscle health remains a central challenge, particularly given the multidimensional nature of muscle function and its interaction across metabolic, neurological, and systemic factors. Studies within this Research Topic highlight a spectrum of approaches, ranging from mechanistically driven metabolomic profiling to pragmatic indices derived from routinely available measures. Collectively, these works underscore both the promise and current limitations of biomarker-driven approaches to muscle health.

At the mechanistic end of this spectrum, Rousseau et al. (5) examined circulating acylcarnitines as predictors of post-ICU muscle recovery. Acylcarnitines are widely used as indirect markers of mitochondrial fatty acid oxidation and metabolic flexibility (23, 24), and disturbed acylcarnitine profiles have been linked to impaired mitochondrial metabolism post-surgery and in critical illness (23, 24). These alterations are consistent with the metabolic dysregulation observed in ill patients, where impaired mitochondrial function and reduced oxidative capacity contribute to systemic energy imbalance and tissue catabolism (25). Despite this strong biological rationale, Rousseau et al. (5) found that short- and long-chain acylcarnitine profiles at ICU discharge were not independently associated with muscle outcomes at three months, and that models incorporating these metabolites failed to predict subsequent muscle health. This disconnect between mechanistic relevance and predictive performance highlights a key limitation of current biomarker approaches: metabolomic signals may reflect underlying pathophysiology but lack specificity to serve as standalone clinical tools, reinforcing the need for multimodal integration of clinical data. In contrast, Hu et al. (3) examined a more pragmatic composite index of muscle health, the muscle quality index, defined as handgrip strength relative to appendicular skeletal muscle mass. Higher muscle quality index was associated with a lower prevalence of urinary incontinence, extending the scope of muscle health beyond traditional mobility and metabolic outcomes, and reinforcing the concept that muscle health is closely tied to broader physiological systems, including pelvic floor function and continent mechanisms (26, 27). While the cross-sectional design limits causal inference, the muscle quality index may be a clinically accessible metric that integrates muscle function and body composition, highlighting the value of composite indices in capturing functional relevance.

Pan et al. (11) further contribute to this theme by evaluating the creatinine-to-body weight ratio as a surrogate marker related to relative muscle mass in a large retrospective cohort analysis. Serum creatinine is primarily derived from skeletal muscle metabolism and, when interpreted relative to body size, may serve as a pragmatic proxy for muscle mass. A lower creatinine-to-body weight ratio was independently associated with a higher risk of incident prediabetes, with evidence of a non-linear relationship and a potential threshold effect below which risk increases sharply. These findings are consistent with a broader body of literature linking reduced muscle mass to impaired glucose metabolism, insulin resistance, and cardiometabolic risk (28, 29). A key strength of this study lies in its scale and analytical approach, including the use of spline modeling to explore non-linearity and comprehensive sensitivity analyses to support robustness. An appeal of this index lies in its scalability and reliance on routinely collected clinical data; however, as an indirect proxy influenced by renal function, diet, and other confounders, it should be interpreted as a broad metabolic marker related to muscle mass rather than a direct measure of muscle tissue.

Taken together, these studies highlight the diversity of approaches to assessing muscle health, reflecting a field that spans mechanistic biomarkers, functional composites, and pragmatic clinical measures. Mechanistically informative markers provide biological insight but may lack predictive utility in isolation, whereas pragmatic indices and surrogate markers offer clinical feasibility but reflect multiple overlapping physiological processes, which can limit their specificity and interpretability. However, several challenges remain. There is substantial heterogeneity in how muscle health is operationalized across studies, limiting comparability and standardization. Moreover, while many measures demonstrate correlational value, few have established predictive validity across diverse populations or responsiveness to intervention. Addressing these gaps requires developing integrated, multimodal frameworks that combine structural, functional, and metabolic indicators to better capture the complexity of muscle health and guide targeted interventions. Within this broader context, understanding how muscle health manifests across specific disease states provides an important next step. Clinical populations offer a unique lens through which the functional consequences of impaired muscle health can be observed, highlighting the role of muscle not only as an outcome of disease but also as a key modifier of disease progression and prognosis.

## 4 Muscle health in specific clinical contexts

Muscle health is profoundly influenced by disease states, where sarcopenia, weakness, and morphological changes both reflect and contribute to disease burden. Increasingly, muscle is conceptualized not merely as an outcome of disease, but as a modifier of disease progression and prognosis, with implications spanning neurological, vascular, and oncological conditions. A substantial body of literature consistently demonstrates that reductions in muscle strength and quality are associated with adverse outcomes across diverse clinical populations, including increased disability, cardiometabolic risk, and mortality (30, 31). Within this theme, the included studies illustrate how muscle impairments manifest differently depending on the underlying pathology, while focusing on functional consequences.

Shin and Lee (10) demonstrated that individuals with both Parkinson's disease and sarcopenia exhibit greater reductions in knee extensor strength and altered contractile properties (e.g., reduced rate of force development) compared to those with either condition alone. These findings align with prior work showing that Parkinson's disease is associated with impairments in neuromuscular activation, strength, and rapid force production, which contribute to bradykinesia and fall risk (32, 33). Importantly, the additive effect of sarcopenia suggests that muscle deficits in neurodegenerative disease are not solely neurologically mediated, but may also reflect peripheral muscular deterioration, reinforcing the need for integrated neuromuscular assessment and intervention strategies. In contrast, Li et al. (4) highlight the role of muscle strength as a protective modifier in cardiovascular health. Their findings indicate that higher grip strength amplifies the benefits of favorable lifestyle behaviors, reducing cardiovascular disease risk substantially in combined models. This is consistent with a growing body of prospective evidence showing that muscular strength is independently associated with reduced all-cause and cardiovascular mortality, even after accounting for traditional risk factors (34). Together, these findings support the concept that muscle strength is not only a marker of health status, but also a contributor to cardiovascular resilience.

Edwards et al. (7) provide an oncology perspective describing how androgen deprivation and related therapies induce sarcopenia-like changes, including reductions in muscle fiber size and increased intramuscular adiposity. These observations are supported by prior clinical and mechanistic research demonstrating that androgen suppression leads to rapid declines in lean mass and strength, contributing to functional impairment and reduced quality of life (35). Importantly, Edwards et al. (7) emphasize that these changes are at least partially modifiable, with resistance training and nutritional interventions shown to attenuate muscle loss and preserve function in cancer populations. Collectively, these studies highlight both shared and disease-specific features of muscle dysfunction. Across neurological, cardiovascular, and oncological contexts, reduced muscle strength and quality consistently emerge as key determinants of functional capacity and clinical outcomes. However, the underlying mechanisms differ, ranging from impaired neural drive in Parkinson's disease to systemic metabolic and lifestyle interactions in cardiovascular disease to therapy-induced catabolic states in cancer, suggesting that interventions must be tailored to the specific pathophysiological context.

Several gaps remain. First, much of the literature, including the studies in this section, is cross-sectional or associative, limiting causal inference. Second, there is a need to integrate mechanistic and clinical outcomes, particularly to understand how changes in muscle morphology and function translate into specific disease states. Third, while exercise and nutritional interventions are consistently proposed, relatively few studies have directly tested disease-specific, precision-based muscle interventions in randomized trials. Future work should therefore focus on longitudinal and interventional designs that clarify whether targeting muscle health can meaningfully alter disease trajectories across these clinical populations.

## 5 Interventions for enhancing muscle health

Translating research into practice requires evidence-based interventions that promote muscle maintenance and recovery. In sarcopenia, recent literature consistently supports exercise as the cornerstone of treatment, with resistance training serving as the primary anabolic stimulus. However, when broader clinical outcomes such as physical performance, balance, and functional independence are considered, multimodal programs that combine resistance, aerobic, and balance training may offer additional benefit (36–40). While resistance exercise remains the most reliable modality for improving muscle mass and strength, combined approaches appear particularly relevant when the therapeutic goal extends beyond isolated muscular outcomes.

Moretti et al. (9) synthesize this evolving evidence base into a clinically actionable framework for primary sarcopenia. Their paper recommends multimodal exercise programs that integrate resistance, aerobic, and balance training, while emphasizing progressive overload, individualization, and adherence as central principles for effective prescription. This approach aligns with recent original research demonstrating the importance of progressive overload for hypertrophic adaptations (41), and the latest American College of Sports Medicine position stand, which reinforces resistance training as foundational for muscle function and performance, while also focusing on adherence strategies (42). Similarly, the Korean Working Group on Sarcopenia concludes that resistance training is essential for improving muscle mass and strength, whereas combined or multicomponent training may be preferable for broader functional outcomes (39).

This distinction is important as sarcopenia is not simply a loss of muscle mass, but a syndrome characterized by weakness, reduced mobility, impaired balance, and increased vulnerability to disability and falls (30). Accordingly, Moretti et al. (9) are persuasive not because they argue for exercise in general, but because they translate the literature into a pragmatic clinical hierarchy: resistance exercise remains foundational, while multimodal programs may better address the multidimensional nature of sarcopenia (9). Supporting this interpretation, recent network meta-analytic evidence suggests that different combinations of exercise and nutrition optimize different outcomes, with combined interventions often outperforming single-modality approaches, depending on the endpoint considered (40).

## 6 Gaps and future directions

While this Research Topic highlights advances in muscle assessment, biomarkers, disease-specific insights, and interventions, several important gaps remain that limit translation into widespread clinical practice. Across themes, a recurring limitation is the relative scarcity of longitudinal, diverse-cohort data, particularly in underrepresented populations, including non-Western ethnic groups and pediatric cohorts. This constraint reduces the generalizability of current findings and limits the ability to define clinically meaningful trajectories of muscle decline or recovery over time (30). A second major gap is the incomplete integration of emerging technologies and multimodal data streams. Although studies in this issue demonstrate progress in ultrasound-based assessment and biomarker identification, the integration of these approaches with artificial intelligence and multi-omics frameworks (e.g., genomics, metabolomics, and imaging) remains relatively underdeveloped. Recent work suggests that combining imaging with molecular profiling may enhance the sensitivity and specificity of muscle health assessment and enable more precise phenotyping of sarcopenia and related conditions (43–45). However, standardized methods, validated analytics and frameworks, and clinically actionable models are lacking. Third, despite strong evidence supporting exercise as a cornerstone intervention, there remains a need for more randomized controlled trials evaluating multimodal and real-world interventions, particularly those combining exercise with nutritional or pharmacological strategies. While emerging evidence suggests that combined interventions may produce greater improvements across muscle mass, strength, and functional outcomes, heterogeneity in study designs and populations limits definitive conclusions (40). Furthermore, many trials are conducted under highly controlled conditions, with comparatively less attention given to long-term adherence, scalability, and implementation in routine clinical settings, despite these factors being critical for sustained impact.

A cross-cutting challenge underlying these gaps is the lack of standardization across measurement approaches, including variability in imaging protocols, biomarker definitions, and operationalization of muscle quality. As highlighted in Sections 2 and 3, this heterogeneity complicates comparisons across studies and hinders the development of standardized diagnostic thresholds and clinical guidelines. Addressing this issue will require coordinated efforts to establish consensus-driven standards for muscle imaging, functional assessment, and biomarker interpretation.

Looking ahead, advancing the field will require more tightly integrated, translational approaches that bridge mechanistic insights and clinical application. Priorities should include (1): validation of promising biomarkers and indices in large, longitudinal cohorts; (2) development of integrated, multimodal assessment frameworks combining imaging, functional, and molecular data; (3) implementation-focused trials evaluating sustainable, real-world interventions; and (4) refinement of phenotype-specific intervention strategies that align with individual variability in muscle health status and disease context. Given the growing recognition of muscle health as a key determinant of aging, metabolic health, and disease resilience, these efforts are essential for moving the field from innovation to equitable and impactful clinical application.

## Data Availability

The original contributions presented in the study are included in the article/Supplementary Material, further inquiries can be directed to the corresponding author.
